# Treatment and monitoring of a high-density population of bare-nosed wombats for sarcoptic mange

**DOI:** 10.1371/journal.pone.0332138

**Published:** 2025-10-01

**Authors:** Tanya N. Leary, Lyn Kaye, Olivia Chin, Kar Yee Phoon, David Phalen

**Affiliations:** 1 Department of Climate Change, Energy, The Environment and Water, New South Wales National Parks and Wildlife Service, Parramatta, New South Wales, Australia; 2 Wildlife Health and Conservation Clinic, Sydney School of Veterinary Science, University of Sydney, Camden, New South Wales, Australia; Beni Suef University Faculty of Veterinary Medicine, EGYPT

## Abstract

*Sarcoptes scabiei* causes a fatal disease (mange) in bare-nosed wombats (BNWs) (*Vombatus ursinus)* across their range and can threaten isolated populations with extinction. Repeated dosing of moxidectin (Cydectin®) at a dosage rate of 0.5 mg/kg is effective at treating individual BNWs but is difficult to administer on a population basis where treatment success has varied. This paper documents the temporary (~20 month) eradication of mange from a semi-isolated population of BNWs using repeated dosing of Cydectin® administered by burrow flaps. Treated BNWs were marked with nontoxic paint and selected burrows were monitored with camera traps demonstrating that 64–96% of wombats in the population were treated with each dosage. Treatment success was attributed to the installation of burrow flaps on all burrows in the treated area. This treatment program shows that isolated high-density populations can be successfully treated for *S. scabiei* infection with repeated dosages of Cydectin® (0.5 mg/kg) and questions the need for higher dosages that have been advocated. Mange returned to the population of BNWs after 20 months possibly as the result of migration of an infected BNW from a nearby population, suggesting mange affected populations may require periodic retreatment. Monitoring of burrow entrances confirmed that burrows provide habitat used by many species of birds, reptiles, and mammals, and suggest burrows could be occasional sites of mange spillover among species. Camera trap monitoring also showed when BNWs in this population leave and return to their burrows; how many BNWs enter a burrow and explore the burrow entrances each night; and how these parameters are impacted by season and mange status; variables that are valuable to know when treating populations of BNW for mange.

## Introduction

*Sarcoptes scabiei* is a mite that causes a highly contagious skin disease, sarcoptic mange, (hereafter referred to as mange). In Australia, mange is an introduced disease that has become widespread and has been recorded in at least ten native and five invasive wildlife species [1–5]. Mange is reported in two of the three wombat species – the common or bare-nosed wombat (BNW) (*Vombatus ursinus*) and the southern hairy-nosed wombat (*Lasiorhinus latifrons*) and occurs across their range [4–9].

In wombats, infection with *S. scabiei* induces a hypersensitivity reaction resulting in an intensely irritating inflammatory skin disease, characterised by crusting, the formation of fissures in the skin and hair loss [10,11]. These lesions and trauma caused by scratching predispose wombats to secondary bacterial and fungal infections [5,6,12–14]. Severely affected animals have increased heat loss, which they are unable to compensate for by increased food consumption, lose weight and many may die within three to four months post-infection [5,9,10,15–17].

Mange can occur in wombat populations across a range of densities and is more commonly observed in areas with agricultural land uses. Both theoretical and empirical literature increasingly demonstrate varied epidemiology, including endemic disease with stable populations and occasional epidemics causing local population collapses [5,18–24]. Transmission of *S. scabiei* between BNWs is believed to occur in the bedding chambers of their burrows as burrows may be sequentially shared or occasionally co-occupied [18,25–30]. In these chambers, mange mites may survive off host for up to 19 days in the cool (10°C) and humid (95% humidity) environmental conditions they provide [31,32] but the duration of their survival may be less in summer [30].

Due to the high public profile of the suffering that mange causes in wombats and fears for its regional and population-wide impacts, animal welfare and community groups have a long history of treating wombats at an individual and, less frequently, the population level using non-invasive methods such as the ‘pole and scoop’ method where a topical mange medication is poured directly onto wombats by a person and the ‘burrow flap’ method where mange medication is spilled onto the first wombat entering or leaving a burrow [4,29,33,34]. One of the most frequently used drugs to treat wombats for mange is moxidectin (Cydectin® - Virbac (Australia) Pty Ltd, Milperra NSW 2214, Australia). Cydectin® is a widely used pour-on anthelmintic approved for use in cattle and red deer. It is popular because it is non-irritating to eyes or skin, its efficacy is not diminished if the fur is wet or if it rains shortly after application [35], and moxidectin has low mobility in the environment [36]. It also has a wide safety margin so the vagaries associated with the administration of the drug to a wild animal that might result in a higher than recommended frequency of administration are not likely to cause toxicity given the most commonly recommended drug dosage rate (0.5 mg/kg to 1.2 mg/kg), delivered once a week for eight to twelve weeks [34,37–40].

Treating populations of wombats is challenging because all wombats in the population must be treated multiple times to kill the mites on them and for mites in the environment that could reinfect them to die off. The pole and scoop method is not feasible as a population-wide treatment as most wombats cannot be approached sufficiently closely to be treated. Also, population-wide treatment is not easily done with burrow flaps as multiple wombats may enter or exit a burrow in one night, wombats can destroy and avoid burrow flaps, finding all wombat burrows is challenging, and Cydectin® rapidly degrades with exposure to sunlight [4,29,41]. Additionally, population scale treatments are labour intensive.

Recently, concerns have also been raised about treatment failures occurring in individual wombats with the topical applications of Cydectin® at the traditional dosage rate (0.5 mg/kg). While there are many potential causes for treatment failure, some have suggested that by increasing the volume of moxidectin applied with treatment [33,42], even to as much as 200 ml (40 mg/kg) per wombat, two to ten times the currently approved dosage [43,44], could improve treatment outcomes [33]. Treating with such high dosages, however, is not without risk and has the potential to cause toxicity in wombats or other animals entering the burrows and may result in significant amounts of moxidectin being released into the environment [45,46].

The overarching aim of this study was to determine the immediate and long-term efficacy of a population-wide treatment for mange using Cydectin® at a dosage rate of 0.5 mg/kg in a high density BNW population. As extensive camera trap monitoring of the population was done prior to, during, and after treatment we were also able to investigate critical BNW behaviours that are valuable when designing a treatment plan for a BNW population with mange. Lastly, we were able to document how other species of animals in the treatment area used wombat burrows and burrow entrances, which is of interest for potentially rare cross-species exposures. For example, swamp wallabies and invasive red foxes are known to utilise wombat burrow entrances and are sporadically reported as suffering from sarcoptic mange, yet there is little research investigating these associations.

## Materials and methods

### Study site

**Fig 1:**
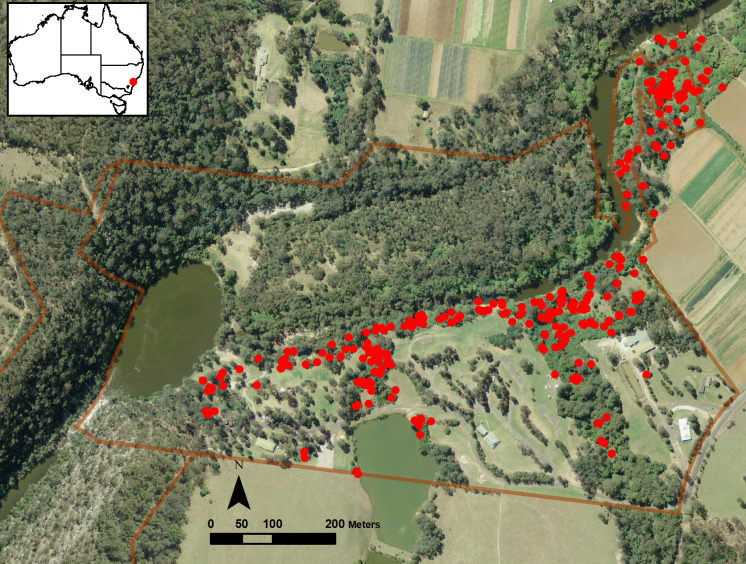
Location of the study site. Red circles indicate wombat burrows treated. (Basemap - © State of New South Wales, Spatial Services, a business unit of the Department of Customer Service NSW, [20/04/25]).

The study took place at Bents Basin State Conservation Area (SCA) (−33.931079˚S, 150.637156˚E), which lies on the south-west edge of the Cumberland Plain, approximately 54 km south-west of Sydney central business district. Bents Basin SCA is a small (50 ha) reserve that includes approximately 1.4 km of the Nepean River, another 0.95 km of subsidiary drainage lines and the 2.1 ha “basin” itself (Fig 1).

The reserve includes grassed areas (~13 ha) that are utilised by visitors for picnics and camping. The other vegetation communities within the Bent’s Basin SCA are predominately threatened ecological communities that are poorly represented on the Cumberland Plain. The BNW burrows are within the River-Flat Eucalypt Forest on Coastal Floodplains of the Sydney Basin Bioregion Endangered Ecological Community (24.5 ha) that is found on the south side of the Nepean River. The northern side of the Nepean River is a frequently inundated floodplain and likely supports few wombats due to the extremely soft sandy soil making burrows prone to collapse.

Bents Basin SCA is adjacent to agricultural/ pastoral land including market gardens, a horse stud, and adjoins Gulguer Nature Reserve (399 ha) which lies in the shale/ sandstone transition zone. The riparian zone is highly dynamic and the majority of the SCA is flood prone. Introduced vines and other weeds are common along the river. Bent’s Basin SCA has a mean rainfall around 660 mm per year, hot summers (mean maximum January temperatures 30.3°C) and mild winters (mean maximum July temperature of 17.5 °C) [47].

The Bents Basin BNW population is regionally significant, being one of the last remaining BNW populations on the Cumberland Plain and the only population within a conservation reserve. The Bents Basin SCA population is a high-density population of at least 50–60 wombats (approximately one wombat per hectare), potentially making it more susceptible to rapid transmission and extirpation by mange [5].

### Monitoring of BNW burrows with camera traps

All aspects of this study were conducted under Scientific Licences SL100349 and SL101462 issued under the New South Wales *Biodiversity Conservation Act 2016* and approved under the Department of Climate Change, Energy, the Environment and Water Animal Ethics Committee under Animal Research Authorities numbers 020404/08 and 141028/01.

### Mange surveillance

Burrows were monitored with camera traps prior to, during, and after treatment. The pre-treatment assessment of mange prevalence in the population was conducted in July 2014 over six nights at 34 burrows. During treatment, burrows were monitored with camera traps for the first eight weeks (T01 to T08 – May/ July 2015), and for two weeks following the last two treatments in October and November 2015 (T12 and T13). To determine whether mange re-emerged in the population, burrows were monitored with camera traps for 2-week periods at one (December 2015 - TP01m), six (May 2016 - TP06m), eight (July 2016 - TP08m), 12 (November 2016 - TP12m), 20 (July 2017 - TP20m) and 41 (April 2019 -TP41m) -months after the final treatment. The number of cameras deployed per week ranged from 21 to 47 dependent on camera and volunteer availability.

Reconyx cameras (HC600 Hyperfire™ no glow Covert Infrared or PC900 Hyperfire™ No glow Covert Infrared; Holman, Wisconsin, USA) made up most of the cameras used. Cameras were programmed with the passive infrared motion detector at the highest level of trigger sensitivity to take three shots per trigger, with the shortest interval between pictures (two frames per second) and the shortest delay (none) between trigger events and at the highest picture resolution and night-time picture quality. Information collected with each image included image number, date, time, and temperature.

Cameras were deployed at burrow entrances deemed to be active. Burrows were considered active if the entrance was clear of vegetation, if there was chewed vegetation around the entrance, or evidence of recent digging activity, footprints, or fresh scats. Cameras were set at variable distances from the burrow, but generally were more than 1.5 m away from the burrow entrance; approximately 1 m above the ground; angled down to face the burrow entrance either at right angles to it or directly in front of it. The treatment area was divided into five similarly sized zones. Approximately, the same number of cameras were deployed in each zone, so that the entire treatment area was monitored each time the cameras were deployed. Efforts were made to select active burrows to monitor with cameras so that they were evenly spaced across each zone and the extent of the study area. This, however, was not always possible due to changes in activity at a burrow, the nature of the terrain and the fact that the wombat burrows were predominately found along the edge of the river and adjacent creeks.

### Camera image processing

On retrieval, camera images were downloaded and all fauna images were identified and tagged using the photo-tagging software Exif-Pro V 2.1.02 [48]. Each series of BNW images beginning from the first field that contained the wombat to when the wombat left the field of view was classified as a camera event. Camera images were defined as a new camera event if there was at least 30 minutes between successive camera triggers, except where the successive triggers were by the same clearly identifiable individual, e.g., a joey waiting at the burrow entrance.

The severity of the mange lesions on each wombat was assessed by determining both the overall mange severity score and the score for the most severely affected segment. The overall mange severity score was based on previously described methods [9,15,16,29]. Each side of the BNW was divided into seven segments – head, shoulder, forelimb, stomach, back, hindlimb and rear/ rump, giving a total of 14 segments per animal [15]. Each body segment was examined for hair loss, skin thickening and skin reddening (daytime photos only) and given a score between 0 and 10. A score of 0 indicated a healthy segment with no signs of mange and a score of 10 indicated severe mange where >70% of the segment was affected by mange. Mange severity was only determined and reported if greater than three segments on one side of a wombat were visualised [13]. Scores from all observed sections of each wombat were averaged to give an overall mange severity score. Given that alopecia is often patchily distributed in wombats with mange, the severity of the mange in each wombat was also estimated by recording the severity of the most affected segment – hereafter referred to as the maximum segment score (sensu 9). Consistent with a previous study [9], we categorised mange severity by maximum segment score, where maximum segment score ≤ 2 was considered healthy; 3 as early mange; 4–6 as moderate mange; 7–8 as severe mange; and 9–10 as late-stage mange. For analyses, where mange severity categories were pooled, we considered healthy animals to have a maximum segment score of ≤ 2 and mange infected BNWs to have a maximum segment score of ≥ 3. Skin lesions on the rump/ rear and caudal-dorsal back can be caused by fighting injuries [49], lacerations made by barbed-wire fences, rubbing against rocks, tree trunks or logs and other yet to be described diseases. Therefore, because otherwise healthy wombats had these lesions, rump/ rear and back segments were not included in maximum segment score. This is consistent with the reported predilection of sarcoptic mites for the anterolateral surface of wombats [13].

### Additional information recorded from each camera event

Except for camera events from the pre-treatment survey in July 2014, each camera event was tagged with a range of other information derived from the photos (Table A in S1 Appendix) and activity of wombats was classified into one or more activity categories – entering or exiting burrow; investigating burrow without entry (standing at opening of the burrow often vocalising, sniffing, urinating or defecating); sitting, standing or sleeping outside of the burrow without investigating the burrow; sunning; sandbathing; social interaction (generally between mother and young, but occasionally between two adults); and passing by without investigating the burrow. We also tagged the photo of the first animal through the treatment flap, i.e., the first animal to lift the treatment flap such that a treatment would be delivered to the head, shoulders or back of the BNW. During post-treatment monitoring, an animal was considered the first animal through the treatment flap if its head and shoulders enter the mouth of the burrow even though no treatment flap was present. Other basic information recorded included number of images per camera event and the duration of camera event (time between first and last image).

### Treatment

Wombats were treated with moxidectin (Cydectin® pour-on for cattle and red deer; Virbac Animal Health Pty Ltd, Milperra, NSW, Australia). Treatment was administered topically using remote treatment stations, hereafter called treatment flaps. Treatment flaps consisted of a modified burrow flap assembly. The burrow flap assembly consisted of a wire frame secured either side or over the BNW burrow entrance with a hinged flap (generally made of the lids of plastic ice-cream tubs – 2 litre or 4 litre) hanging down from the frame similar to that depicted in Martin et al. [29] but having two reservoirs for fluids. As a BNW entered or exited the burrow, the flap lifted and deposited the contents of the reservoirs onto the BNW. The two reservoirs fitted into each flap were plastic bottle caps (approximately 40 mm in diameter each containing 4 ml of fluid). One bottle cap administered a 4 ml dose of Cydectin®, and the second administered a 4 ml dose of black nontoxic face and body paint (Derivan Pty Ltd, Rhodes, NSW Australia) that was detectable in images taken by the infra-red cameras. At burrows with wide entrances, it was necessary to either install wider flaps made of Corflute (Corax, Melbourne, Victoria, Australia), with double the number of bottle tops or to install multiple flaps.

During the pre-treatment investigations, wombats were seen to avoid burrows with treatment flaps for at least 3 to 4 days after installation and during that time reactivated burrows that had been deemed to be inactive. Consequently, during the treatment phase of this study, all known burrows (not just those that were deemed active) were provided with treatment flaps.

Burrows were treated 13 times between 30 May 2015 and 14 November 2015. Eight doses were delivered at weekly intervals (T01 – T08), followed by two at fortnightly intervals (T09 - T10), and then three doses at monthly intervals (T11 – T13). On the day of each treatment, the area was searched and burrows that may have been previously overlooked, re-activated or recently dug “shallow burrows” were identified. If the burrow was “available” to a BNW, i.e., wasn’t flooded or blocked by debris, weeds, landslides or the entrance collapsed, a treatment flap was added. If a burrow was “unavailable” but visible or a shallow burrow, it was monitored during subsequent treatment periods, and a treatment flap was placed on the burrow when it became “available” or when a shallow burrow was enlarged to the extent it could be utilised by BNW. At the beginning of each treatment period, treatment flaps that had been damaged were replaced. We used approximately 9,500 ml of Cydectin® over the course of the treatment period costing approximately $2,000 Australian dollars.

Preliminary trials prior to treatment showed that paint markings are visible for up to 14 days but most distinct in the first 4–5 days after application. To provide an index of the proportion of the population that was treated each week, the proportion of camera events that contained individuals marked (either strongly or faintly) with paint was noted during the first 4 days of each treatment week. This was not possible for treatment T13 as not all reservoirs were refilled with paint.

### Mange prevalence, severity, and potential for transmission

The proportion of camera events in five health categories (healthy; early mange; moderate mange; severe to late-stage mange; unknown = fewer than three segments visible or image quality too poor to determine mange status) based on maximum segment scores pre-treatment (34 active burrows monitored) were compared with the first treatment (32 active burrows monitored) as an index of mange prevalence across the population. The proportion of burrows in each of three categories: (a) used by only healthy BNWs, b) used by only mangy BNWs and c) used by healthy BNWs after occupation by mangy BNWs was compared between July 2014 (pre-treatment) and May/ June 2015 (T01) as an index of the potential for mange transmission. The significance of difference in frequency distributions between pre-treatment and T01 were determined using separate Chi-squared tests.

Overall mange severity score of all BNWs camera events was used as an index of population-wide mange severity during treatment and post-treatment monitoring and as an index of resolution of mange signs with treatment. Although it was possible to distinguish some BNWs with distinctive patterns of hair loss or other features, it was not possible to distinguish all individuals photographed and therefore some individuals are likely to have been scored for mange severity multiple times per day or per treatment week. Consequently, we did not statistically analyse overall mange severity score for each treatment or the population-wide maximum segment score of the animals photographed being treated and instead present this data graphically. However, we compared separately the frequency distribution of mange severity categories at T01 and T13, from all BNW camera events, and for the animals photographed being treated (to minimise potentially double counting individuals) using Kolmogorov-Smirnov two sample tests, to determine the success of treatment in resolving mange symptoms. We also correlated maximum segment score of the first animal through the treatment flap to investigate whether maximum segment score decreased with successive treatments.

We calculated the mean number of flap interactions (the number of times the treatment flap was lifted such that a treatment would have been administered if still present in the reservoir) per night per active burrow during treatment (T01 – T13) to further examine the potential for mange transmission by both temporary visitors to the burrows and BNWs that occupied the burrow by day, and to investigate the potential for multiple treatments to be delivered per night if, in the future, a multidose application method could be used. In addition, we compared separately the frequency distribution of flap interactions and the frequency distribution of burrow investigation without burrow entry, with the time of day (in hourly increments) in winter, and summer using Kolmogorov-Smirnov two sample tests. We pooled four weeks of monitoring data from winter (T01, T02, T03 and T04) when both healthy and mange infected animals were present and compared with four weeks of monitoring data in summer (T13 and TP01m) when only healthy animals were present. We also compared frequency distribution of flap interactions in winter when only healthy animals were present (TP06m and TP08m) and when healthy and mange infected animals were present (T01 to T03) to determine whether presence of mange infected animals altered the frequency distribution of burrow entries/ investigations (flap interactions). We graphed frequency distribution, to determine the time of day when most entries/ investigations occurred, and whether there was a period of peak activity at the burrow entrance.

### Assessing the behavioural impacts of mange

#### Scratching.

We explored whether mange infected BNWs behaved differently to healthy BNWs by determining whether mange infected animals scratched more frequently than healthy BNWs. We separately compared the ratio of camera events that recorded BNWs scratching and “not scratching” in T01 and T02 pooled, and for all monitoring periods pooled (to increase the sample size), between healthy and mange infected animals using separate Chi-square tests. We calculated the proportion of camera events that recorded BNWs scratching in each treatment period. Simple linear regressions were used to explore the relationship between irritation (scratching) and the number of treatments (during the treatment phase only) and between irritation and the number of months after the final treatment (T13 – TP41m).

#### Diurnal activity.

We used log-linear analysis to examine the association between mange severity category and the time of day of activity at the burrow in treatment periods T01 to T04 pooled. Time of day of activity was categorized in two ways: 1) in two time periods – nocturnal (sunset to sunrise) and diurnal (sunrise to sunset); and 2) four time periods – early evening (sunset to midnight), pre-dawn morning (midnight to sunrise), morning (dawn to midday) and afternoon (midday to sunset). The log-linear analysis is similar to the analysis of two-way contingency tables, but the relationship is analysed by taking the natural logarithm of the counts within each cell [50,51].

#### Time of daily emergence and return to burrows.

We examined whether mange significantly influenced the time of return to the burrow and emergence from the burrow each day relative to sunrise and sunset respectively during treatments T01 to T04 using separate one-way Analysis of Variance (ANOVA). The time of return to burrow comparison used all mange status categories (healthy, early, moderate, severe and late-stage mange), but the time of emergence comparison pooled BNWs with late-stage and severe mange into one category, as we recorded few late-stage mange affected BNWs emerging.

Time of return to the burrow was calculated as minutes before sunrise and time of emergence was calculated as minutes after sunset, using the time stamp of the last photo in each camera event. Consequently, time is expressed as a negative number for animals that returned to the burrow after sunrise or emerged from the burrow before sunset. The daily sunrise and sunset times were estimated using data for Wallacia New South Wales [52] which is approximately 7 km north of Bents Basin.

In some cases, BNWs made multiple foraging bouts in an evening (particularly females with dependent young). Where multiple foraging bouts were made, we only included the time of first emergence from the burrow and the time of the final return to the burrow in the analysis. We also excluded the time of exits and re-entry to the burrow of animals that sunbathed, sandbathed or rested temporarily in front of the burrows.

To explore whether temperature influenced time of emergence or return to the burrow, during treatment T01 to T04 when both healthy and mange affected animals were present in the population, we correlated separately ambient temperature recorded by the camera at the burrow at time of emergence or at time of return to the burrow with the maximum segment score (mange severity score). We explored whether the temperature at the burrow differed between BNWs that were healthy, or had early, moderate, severe, or late-stage mange using a one-way ANOVA. Post-treatment, when all BNWs were healthy, we also explored whether there were seasonal differences in emergence time (relative to sunset) and return to burrow time (relative to sunrise) between summer (TP01m) and winter (TP06m and TP08m pooled) using a Student’s t-test.

#### Burrow switching.

To determine whether mange affected animals switched burrows more frequently than healthy BNWs, we calculated the number of consecutive days a burrow was occupied per week by mange infected and healthy BNWs during treatment periods T01 to T05 using a Welch two-sample t-test (unequal variance). Within each monitoring week, we were generally able to distinguish if it was the same animal occupying the burrow from a combination of paint marking and distinctive features such as hair loss patterns, ear tears or old fighting scars. However, in a small number of cases, we could not conclusively determine if the wombats photographed at the burrow entrances were the same animal and we have assumed that occupation of the burrow on consecutive days was by the same BNW. Cameras were often moved each week during treatment, so it was therefore not possible to calculate burrow occupancy for more than one week for some burrows.

We examined the number of consecutive days burrows were occupied post-treatment (when all BNW were healthy) during monitoring periods when cameras were deployed for 14 days (TP06m and TP12m) to ascertain the influence of number of days of camera monitoring. During these monitoring periods all BNW were healthy and were not marked, so we were rarely able to distinguish between individuals, but we have assumed consecutive days of occupancy were by the same individual.

### Reproduction – pouch young and joeys-at-heel

As an index of reproductive activity, we classified camera events of females with young into three categories: 1) females with large pouch young – bulge obvious, with or without pouch wear; 2) females with small joeys-at-heel that were less than one-third of the size of the mother and had a narrow muzzle with ears that generally appeared too large for the head, which we estimated to be around 9 to 11 months old [53]; and 3) females with a large joey-at-heel that were more than one-third of the size of the mother and had a broad muzzle and ears proportional to the size of the head, which we estimated to be approximately 12–18 months old. Large joeys-at-heel were often photographed independently of their mother at or near the natal burrow entrance.

BNW birth may occur at any time of year [54], but seasonal peaks in births have been described in some areas [53,55]. We therefore utilise data only from late autumn/ winter monitoring periods to examine reproductive output as it is unknown if there are seasonal birth peaks at Bents Basin. We graphically present data in three two-week monitoring periods: a) treatment period T01 and T02 (May/ June 2015) when mange was prevalent in the population; b) six-months post-treatment (TP06m – May 2016); and c) 20-months post-treatment (TP20m – July 2017). Individuals could generally be distinguished over a two-week period, by features on either the mother or the joey (including the size of the joey) or a combination of features of both.

### Other species photographed at burrows

We quantified the number of camera events of other species photographed at the burrow entrance and noted any species that spent time within the burrow to provide additional information on the benefits that wombat burrows might provide to other wildlife species and identify other species potentially at risk of pathogen exposure. Images of other mammalian species using wombat burrows or in proximity to their entrances were also examined for evidence of mange-like skin lesions.

### Effort

Delivery of the treatment and monitoring program involved six NSW NPWS and two University of Sydney staff, over 215 community volunteers, 42 University of Sydney students and eight contractors. We documented the number of hours required to deliver the pre-treatment assessment, treatment and post-treatment monitoring as well as initial tagging of images. Delivery of treatment and camera monitoring (pre-treatment, during treatment and post-treatment) involved approximately 4,382 person hours. Including initial photo-tagging and data entry, delivery of the overall program was more than 5,994 person hours. However, this effort does not include additional time spent analysing images by NPWS staff to ensure consistency in mange severity scores, identifying mother and young combinations, flap interactions and to collect other behavioural data from camera images.

### Statistical analysis

All means are reported ± 1 standard deviation. All proportional data were arcsine square-root transformed to improve normality [56]. Correlations with maximum segment scores used Spearman’s Rho correlation coefficients as this was treated as ordinal data and all other correlations used Pearson’s Product Moment correlation coefficients.

All statistical analyses (except log-linear analysis) were performed using R Statistical Software (R: The R Project for Statistical Computing (r-project.org)) version 4.3.1. 95% confidence interval (95% CI) for Spearman Rho correlation coefficients were calculated in the R package “RVAideMemoire” (https://CRAN.R-project.org/package=RVAideMemoire). Levene’s tests for homogeneity of variance utilised the R package “CAR” (https://CRAN.R-project.org/package=car). When significant (p < 0.05) results were obtained from ANOVAs, post-hoc pair-wise comparisons were made using Least Significant Differences (LSD) test (α = 0.05) to identify where the differences occurred utilising the R package “agricolae” (https://CRAN.R-project.org/package=agricolae). Log-linear analysis was performed with SPSS Statistics for Windows, Version 17 [57].

## Results

### Pre-treatment

#### Mange prevalence and severity and potential for transmission prior to the onset of treatment.

**Fig 2:**
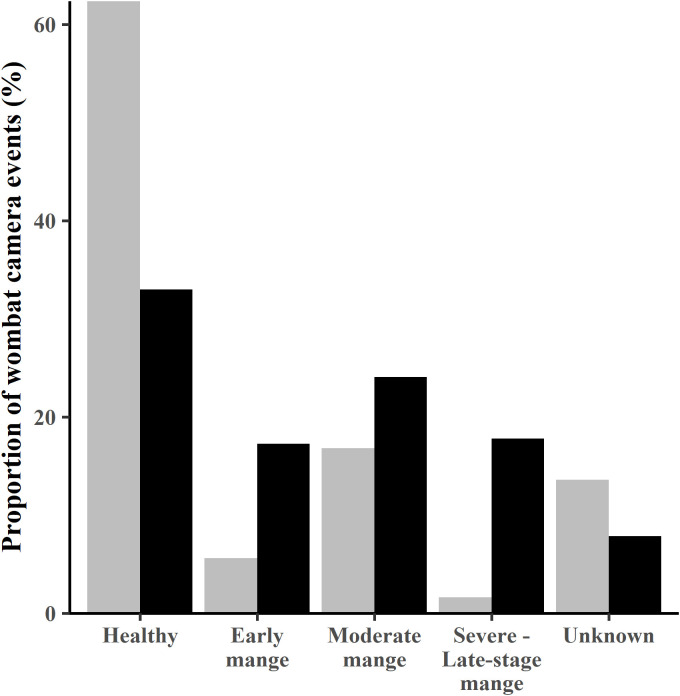
Proportion of camera events by mange severity category pre-treatment and the first week of treatment. Pre-treatment (July 2014) – grey bars; first week of treatment (May/ June 2015) black bars. Unknown – mange status could not be determined from the photos as either the photo was of poor quality, or less than three segments of the animal was visible. Camera events of BNWs with severe and late-stage mange infections are pooled to increase sample size.

Between July 2014 (pre-treatment) and May/ June 2015 (T01), the proportion of camera events recording infected animals (all categories pooled) increased from 24% to 59%. The overall frequency distribution of each mange severity category was significantly different at treatment T01 compared to pre-treatment in July 2014 with proportionally fewer healthy and more mange infected animals recorded at T01 (χ^2^ = 44.5522, 4 df, p < 0.0001) (Fig 2).

The potential for mange mite transmission at burrows increased between July 2014 and commencement of treatment in May/ June 2015, with a decrease in the proportion of burrows recording only healthy BNWs from 49% to 17%, and an increase from 32% to 63% of burrows that were used by a healthy BNW following occupation by a mange infected BNW (χ^2 ^= 9.1832, 2 df, p = 0.01014).

### Treatment and post-treatment monitoring

#### Cameras deployed and images captured.

We deployed cameras to burrows 521 times during treatment and post-treatment monitoring, however, 61 cameras (11.7%) failed or were set so that they did not effectively capture the burrow entrance and were excluded from further analysis. Each monitoring session between 21 and 39 cameras were “effectively” deployed at burrows such that they adequately framed the burrow entrance. The mean number of “effective” cameras did not differ between treatment 28.8 ± 6.4 and post-treatment monitoring 28.8 ± 5.3 (Students t-test: t = 0.010711, 14 df, p = 0.9916). Across treatments T01 to T13 a total of 113 burrows were monitored with cameras for at least one week.

In total, during treatment and post-treatment monitoring, we captured 224,919 images over 4,452 effective camera monitoring nights. Of these, 78,678 were images of BNWs. This equated to 4,708 camera events, of which 3,346 could be scored for mange. The number of images per camera event ranged from 1–909. The majority (94.4%) of camera events comprised of ≤ 50 images. Longer camera events were generally either of animals digging without break or of dependant joeys resting near the burrow entrance awaiting their mothers' return or of joeys interacting with their mothers. In addition, three mange infected BNWs (two late-stage and one with early mange) were photographed sunbaking and sandbathing near the burrow entrance during the daytime for up to 5 hrs and 50 minutes. The duration of a camera events ranged from 1 second to 8 hrs 36 minutes, but only 2.1% of camera events were of greater than 30 minutes duration.

#### Burrows treated and proportion of animals marked/ treated.

With each treatment it was necessary to replace between 3.5% and 17.9% of treatment flaps because they had been destroyed by BNWs and adjust a further 0.7–4.3% of flaps displaced or being avoided by BNWs.

The number of ‘known available’ burrows treated each treatment period increased from T01 (112 burrows) to T13 (210 burrows). The total number of burrows monitored across T01 to T13 (including ‘unavailable’ burrows or shallow burrows) was 256. The proportion of available burrows treated each week ranged from 82% to 100% and at all but two treatment sessions (T05 and T12) we treated greater than 90% of available burrows. The number of burrows effectively monitored with cameras each week ranged from 21–39; or between 12% and 33% of known and ‘available’ burrows each treatment (except T09 – T11 when we did not deploy cameras). Camera monitoring indicated that animals generally received no more than one dose of Cydectin® at a time from the larger Corflute flaps, and that some minor off-wombat spillage occasionally occurred.

**Fig 3:**
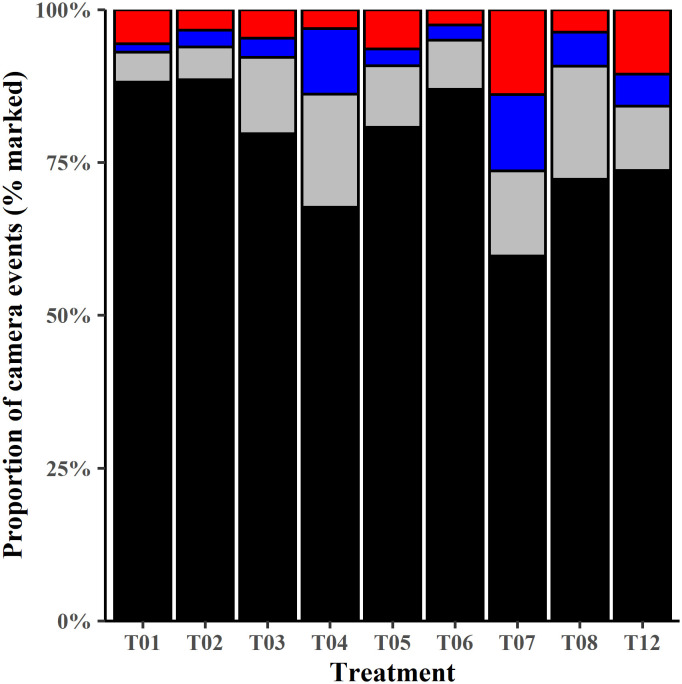
Proportion of camera events showing BNWs marked and unmarked during treatment. Darkly marked BNWs (black fill), faintly marked (grey fill), uncertain if marked (e.g., dorsum or top of head not clearly visible in photographs) (blue fill) and unmarked (red fill). Treatments shown are T01 to T08 and T12. Burrows were not monitored with cameras during T09 – T11 and paint reservoirs were not refilled at T13. Data is pooled for the first four days of each treatment week.

BNWs were marked in more than 89% of the camera events in each treatment (except T07 and T12) (Mean 89.4 ± 6.7% of camera events; range 74–96%; Fig 3). If faintly marked animals were marked at a previous treatment, then we treated between 60–89% of animals (Mean 77.9 ± 9.7%) at each treatment (Fig 3).

#### Mange prevalence and severity.

**Fig 4:**
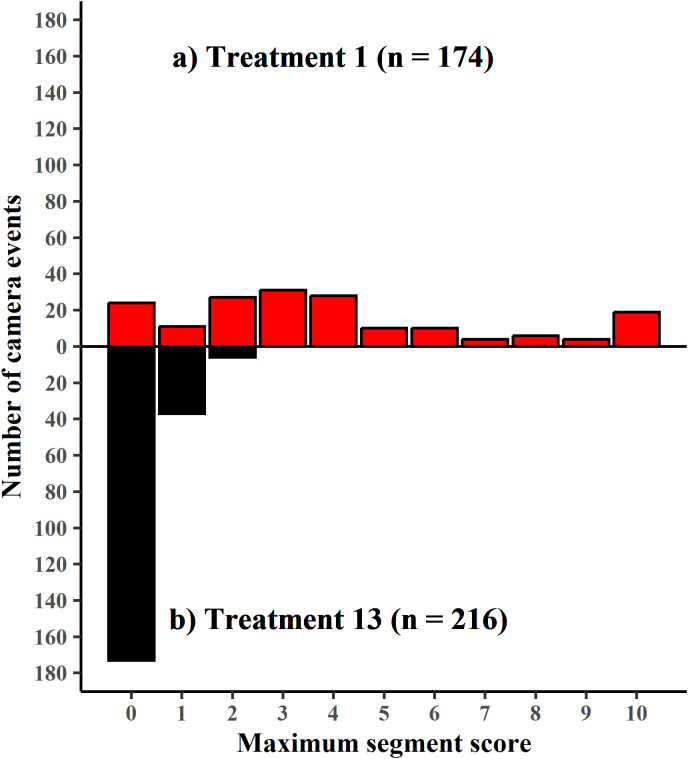
Frequency distribution of maximum segment scores. a) Treatment 1 (upper panel – red bars) and b) Treatment 13 (lower panel – black bars). n = the number of camera events. Only the first week of camera monitoring is used at Treatment 13.

The number of segments scored for mange severity per camera event ranged from three to 14. The frequency distribution of maximum segment scores was significantly different between T01 and T13 (Kolmogorov-Smirnov *D* = 0.77107, p < 0.001, Fig 4), so we did not statistically analyse this data further.

**Fig 5:**
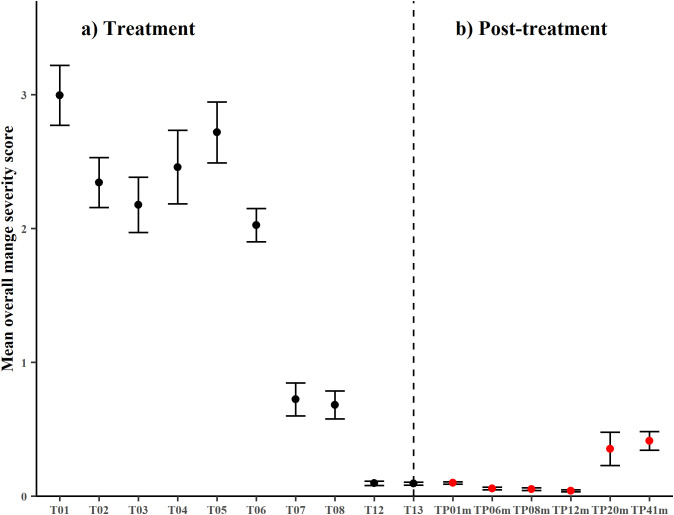
Mean overall mange severity score. a) Treatment (black circles) and b) Post-treatment (red circles). Error bars are ± 1 standard error. All BNW camera events are pooled for each monitoring period.

The population-wide overall mange severity score during the treatment phase fell from 3.0 ± 2.94 at treatment T01 to 0.09 ± 0.23 at treatment T13, with the lowest overall mange severity score at TP12m 0.04 ± 0.11 (Fig 5). The population overall mange severity score initially decreased during treatment (T02 and T03) but rose in T04 to T05. One potential reason for these increased scores was that the most eastern section of the riverbank could only be fully accessed for treatment at T04. By treatment T07 and T08, most animals had resolved all signs of mange or were clearly recovering (individuals with signs of hair growth and/ or no longer appeared emaciated). From T12 through to TP12m, overall mange severity scores were close to 0. At T12 there was a single animal with a maximum segment score of 3 which was a recovering animal, but by T13 no animals had a maximum segment score of ≥ 3 and the maximum segment score of all animals remained below 3 until 20-months post-treatment. The first evidence of re-emergence of mange was at TP20m, when a single emaciated individual with severe to late-stage mange was photographed at three different burrows. This animal was subsequently captured and humanely euthanised. At forty-one months post-treatment, mange had clearly returned to the population with 22 camera events at 9 burrows recording maximum segment scores of ≥3 (range 3–7).

**Fig 6:**
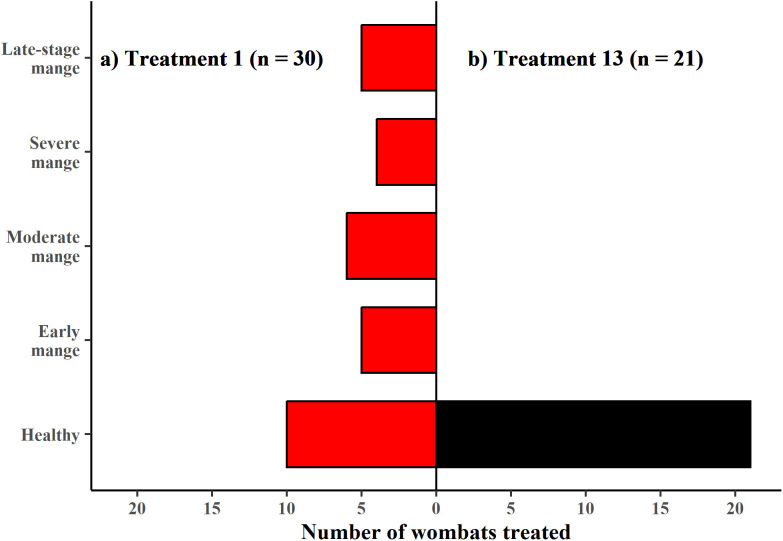
Number of individuals photographed being treated in each mange severity category. Mange severity category is based on maximum segment score. n = number of effective cameras in each treatment period. Only the first week of camera monitoring is used in T13.

The frequency distribution of maximum segment score differed between T01 and T13 of the animals photographed being treated (i.e., the first animal through the treatment flap at each burrow) (Kolmogorov-Smirnov *D* = 0.70476, p < 0.001). All animals treated at T13 were healthy (Fig 6).

The mean maximum segment score of the first animal through the flap showed a similar trend to mean population-wide overall mange severity score and fell between treatment T01 4.4 ± 3.24, and the final treatment T13 0.33 ± 0.66 (Fig A in S1 Appendix). There was a significant negative correlation between maximum segment score and number of treatments (Spearman’s rho correlation coefficient = − 0. 4134059, p < 0.001, 95% CI: −0.5099941, −0.3114857), suggesting mange signs were being progressively resolved with successive treatments.

### Flap interactions and burrow investigations

During treatment (T01 to T13), the number of flap interactions per day at active burrows ranged from 1–69. Thirty-eight percent of all camera events involved BNWs either entering or exiting the burrow or partially entering/ exiting the burrow such that a treatment would be delivered had the treatment flap been full of medication. Whilst it was not possible to confidently differentiate animals visiting each burrow, at one burrow we were able to differentiate at least 19 different BNWs (based on a combination of hair loss, fighting scars and paint marking) that visited (not necessarily entered) a burrow in a single day.

**Fig 7:**
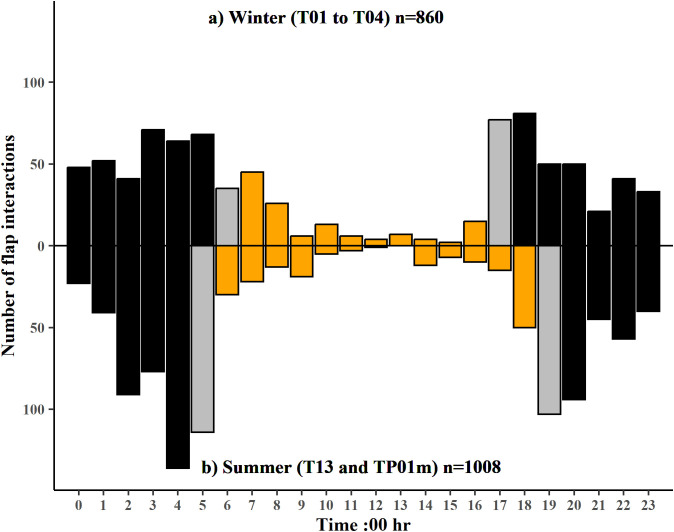
Frequency distribution of flap interactions with time of day in winter and in summer. Data is 4 weeks of monitoring pooled in each season. a) Upper panel – winter (T01 to T04) and b) Lower panel – summer (T13 and TP01m). Bars represent the number of flap interactions in one-hour increments and n = the total number of flap interactions recorded. Orange bars represent flap interactions during daylight hours, grey bars are the hour increment in which sunrise or sunset occurred and black bars are night-time interactions.

The frequency distribution of flap interactions with time of day differed between winter (T01 – T04) and summer (T13 and TP01m) (Kolmogorov-Smirnov *D* = 0.10957, p < 0.001). Flap interactions had a flatter night-time distribution in winter compared to summer which had more pronounced peaks around dawn and dusk. BNWs in both winter and summer investigated more burrows by entering or partially entering burrows within two hours of sunrise and two hours of sunset (Fig 7).

Frequency distribution of flap interactions in winter did not differ between times when both mange affected and healthy animals were present (T01 – T04) and when only healthy animals were present (TP06m and TP08m) (Kolmogorov-Smirnov *D* = 0.074852, p = 0.06669). Frequency of flap interactions in winter by BNWs with and without mange, were relatively evenly distributed across the night with minor peaks before sunrise and after dusk.

Frequency distribution of camera events of burrow investigation without entry to the burrow differed between winter (T01 – T04) and summer (T13 and TP01m) (Kolmogorov-Smirnov *D* = 0.17443, p = 0.02656) (Fig B in S1 Appendix). Distribution of summer burrow investigations without burrow entry had more pronounced peaks around sunrise and sunset while in winter the frequency distribution was flatter but with a minor peak between midnight and one am.

### Behavioural impacts of mange

#### Scratching.

**Fig 8:**
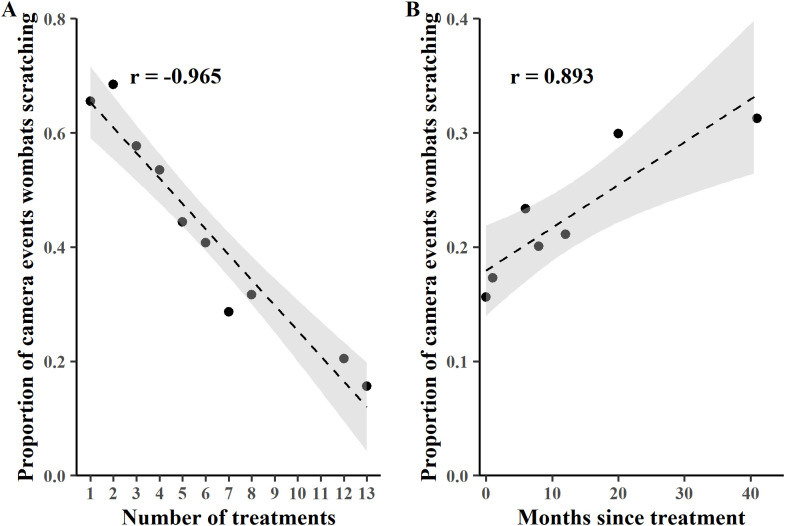
Proportion of camera events recording wombats scratching. A – Left panel shows the relationship between scratching and the number of treatments. Treatments T09 to T11 were not monitored with cameras. B – Right panel shows the relationship between proportion of camera events recording scratching and the number of months after the final treatment T13. Treatment T13 is shown as 0-months post-treatment. All proportional data is arcsine-square root transformed to improve normality. Grey shading represents the 95% confidence interval in both panels.

Both healthy and mange affected animals were photographed scratching. The ratio of BNWs scratching versus “not scratching” captured in camera events at treatment T01 and T02 differed between healthy individuals (22.9% scratching) and mange infected BNWs (52.9% scratching) (χ^2^ = 39.017, 1 df, p < 0.001). When all monitoring periods were pooled the proportion of healthy BNW camera events depicting scratching (7.5%) was similarly significantly lower than the proportion of mange infected BNW camera events that depicted scratching (33.8%) (χ^2^ = 356.78, 1 df, p < 0.001). Scratching BNWs as a proportion of all BNW camera events decreased with successive treatments (t = −10.407, 8 df, Pearson’s product-moment rho = −0.9649961, 95% CI: −0.9919354, −0.8546346; p < 0.001, Fig 8A), but increased with number of months after the final treatment (t = 4.4475, Pearson’s product-moment rho = 0.8934349, 95% CI: 0.4290354, 0.9842687; p = 0.006718, Fig 8B).

#### Diurnal activity.

Only 8.2% of camera events across all treatment and post-treatment monitoring sessions were diurnal and most of this was close to sunrise and sunset. During T01 to T04, there was significantly more nocturnal activity (576 camera events at burrows) than diurnal activity (80 camera events) across all health classes (Table 1; log-linear χ^2^ = 4.313, 1 df, p = 0.038). The significant nocturnal/ diurnal interaction with mange infection (healthy versus all mange classes pooled) can be interpreted as mange affected animals exhibited proportionally more diurnal activity (15.0%) than healthy BNWs (9.7%) (log-linear χ^2^ = 4.313, 1 df, p = 0.038) during T01 to T04.

**Table 1:** Contingency table of number of nocturnal and diurnal camera events with mange severity for treatment periods T01 – T04 pooled.

	Mange Severity				
Time Period	Healthy	Early Mange	Moderate Mange	Severe Mange	Late-stage Mange
***Nocturnal***					
**Sunset – Midnight**	150	42	45	11	30
**Midnight – Sunrise**	166	44	57	18	13
***Total Nocturnal***	316	86	102	29	43
***Diurnal***					
**Sunrise – 12 noon**	33	3	9	1	11
**12 noon – Sunset**	1	2	5	3	12
***Total Diurnal***	34	5	14	4	23
***Grand Total***	*350*	*91*	*116*	*33*	*66*

**Fig 9:**
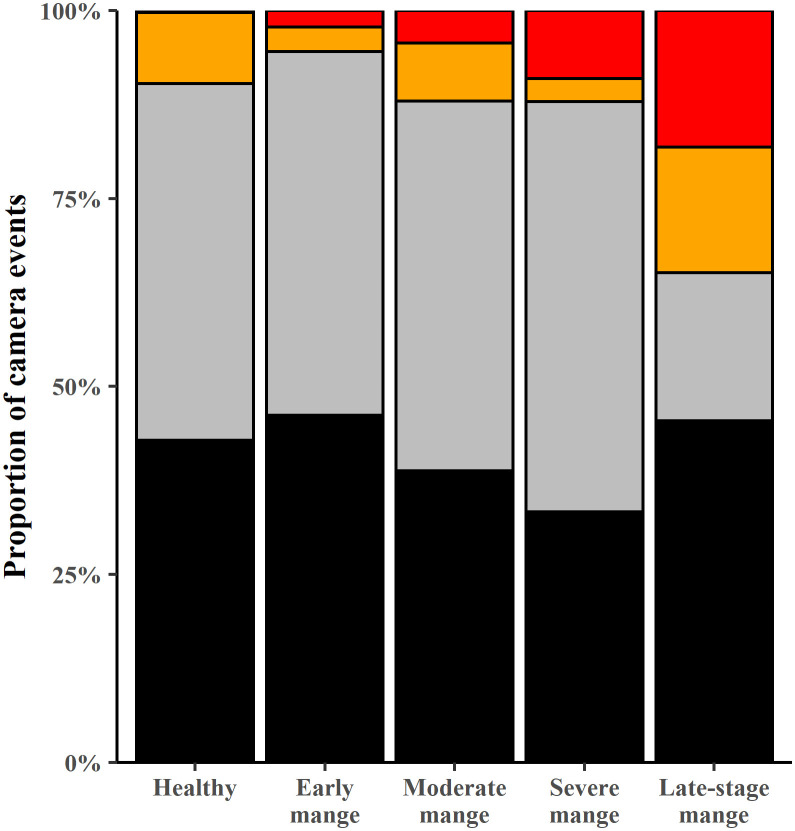
Proportion of camera events of animals in each mange class by time of day. Treatment periods T01 – T04 shown. Between: sunset and midnight (black fill), midnight and sunrise (grey fill), sunrise and midday (orange fill) and midday and sunset (red fill).

When time period of activity and mange severity category were examined in more detail, we found a significant interaction between the time period a BNW was active and the mange severity category (log-linear χ^2^ = 76.437, 12 df, p < 0.001) (Table 1 and Fig 9). The proportion of camera events from midday to sunset increased with mange severity (Fig 9) – 0.3% for healthy; 2.2% early-mange; 4.3% moderate-mange, 9.1% severe-mange; and 18.2% late-stage-mange. The proportion of overall diurnal activity was similar for most health categories (healthy 9.7%, early 5.5%, moderate 12.1% and severe 12.1%) except BNWs with late-stage-mange (34.9%). Proportionally less activity at burrows was recorded between midnight and dawn of BNWs with late-stage-mange (19.1%) compared to healthy (47.4%), and all other mange severity categories (range 48.4–54.6%) (Fig 9).

#### Time of emergence and return to burrows.

Across all monitoring (treatment and pre- and post-treatment), the majority (71%) of returns to the burrow each day were within a four-hour window of sunrise (3 hrs before and one hour after sunrise) for both healthy and mange affected animals. However, both healthy and mange affected animals returned to (and remained within) the burrow outside of this four-hour period.

Mange severity influenced time of return to the burrow each day during treatments T01-T04 (One-way ANOVA F_4, 95 _= 3.104, p = 0.019). Post-hoc analysis indicated that BNWs with late-stage mange returned significantly (Least Significant Difference (LSD) p < 0.05) later (−46.6 ± 246 minutes before sunrise, i.e., 46.6 minutes after sunrise) than healthy (64.4 ± 128 minutes before sunrise), or BNWs with early- (131.4 ± 120 minutes before sunrise), moderate- (103.1 ± 149), and severe- (141.5 ± 133) mange.

The maximum segment score of the BNW was weakly positively correlated with ambient temperature at the burrow when BNWs returned each day during T01 – T04 but was not significant (Spearman’s rho correlation co-efficient = 0.1840942, 95%CI: −0.02459872, 0.36297157; p = 0.06673) but ambient temperature at time of return to the burrow differed between BNW mange severity categories (F_4, 95_= 4.029, p = 0.00462). Post-hoc analysis revealed that the temperature was significantly warmer at the time of return of BNWs with late-stage mange (10.2 ± 5.5 °C) than at the time of return of healthy BNWs (6.3 ± 3.2 °C), or BNWs with early- (4.9 ± 2.5 ˚C), moderate- (5.7 ± 3.0 °C) and severe- (6.0 ± 3.6 °C) mange.

In contrast to returns to burrows in the morning, emergence (exits) from burrows in the afternoon during T01 – T04 were tightly clustered around sunset with 87.2% of all exits recorded within 90 minutes of sunset. The mean emergence time with respect to sunset, did not differ between healthy BNWs (54.3 ± 39.9 minutes after sunset), or BNWs with early- (33.0 ± 18.4 mins), moderate- (40.8 ± 69.4 mins) or severe to late-stage mange (40.6 ± 47.4 mins) during T01 – T04 (F_3, 66_ = 0.761, p = 0.52). Maximum segment score was weakly positively correlated with temperature at the burrow at time of emergence during T01 - T04 but this was not significant (Spearman’s rho correlation co-efficient = 0.2242243, 95%CI: −0.03917206, 0.45489214; p = 0.06203) and ambient temperature at time of emergence from the burrow did not differ between BNW mange severity categories (F_3, 66 _= 1.253, p = 0.298).

Post-treatment we found no difference in time of return (relative to sunrise) of healthy BNWs between autumn/ winter (81.5 ± 128 mins before sunrise) and summer (71.3 ± 96.1) (Student’s t-test t = −0.54683, 162 df, p = 0.5852) nor in the time of emergence from the burrow (relative to sunset) between summer (37.1 ± 37.3 mins after sunset) and autumn/ winter (41.6 ± 28.3 mins after sunset) (Student’s t-test t = −0.8525, 155 df, p = 0.3953), despite significantly different maximum daily temperatures between summer (29.5 ± 3.8 °C) and autumn/ winter (22.2 ± 3.7 ˚C) (Student’s t-test t = 6.9801, 50 df, p < 0.001).

#### Burrow switching.

In treatment periods T01 - T05, BNWs with mange changed burrows more frequently than healthy animals. The mean number of consecutive days per week burrows were occupied differed between healthy BNWs (2.2 ± 1.7 days; range 1–7 days; n = 62 wombat occupations) and mangy BNWs (1.6 ± 1.3 days; range 1–6 days; n = 58 wombat occupations) (Welch two sample t-test t = 2.38888, p = 0.01856, 113.24 df). However, this may be an artifact of the seven-day monitoring period because at six- and 12-months post-treatment (TP06m and TP12m) when burrows were monitored for 14 days and all animals were healthy, burrows were occupied for 4.2 ± 3.4 days (range 1–14).

### Reproduction – pouch young and joeys-at-heel

**Fig 10:**
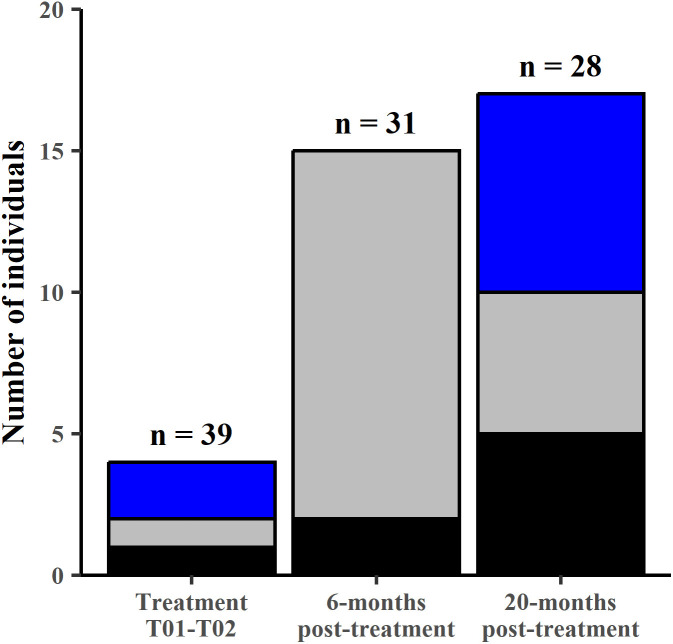
Number of large pouch young and young-at-foot during treatment and post-treatment. Treatment T01 – T02 (June 2015), six-months post-treatment (TP06m – May 2016) and 20-months post treatment (TP20m – July 2017). Bars represent number of individuals of large pouch young (black fill), small joeys-at-heel (~ 8–11 months old) (grey fill) and large joeys-at-heel (~12–18 months old) (blue fill). n = number of active burrows effectively monitored with cameras.

There were signs of increased reproductive output after treatment. Few pouch-young and joeys-at-heel were recorded at burrows at the commencement of treatment (T01 – T02: n = one large pouch young; one small joey-at-heel (~8–11 months old); and two large joeys-at-heel (~12–18 months old). In contrast, at six-months post-treatment we recorded two large pouch young and 13 small joeys-at-heel, and at 20-months post-treatment five large pouch young and five small and seven large joeys-at-heel were recorded (Fig 10). In treatment periods T01 - T02, three of the mothers were healthy and one showed inconclusive signs of early mange. Post-treatment all adult females appeared healthy. No joeys showed signs of mange at any time during the study.

Smaller joeys-at-heel were mostly cached at the burrow (95% of camera images of small joeys) and were most frequently photographed outside the burrow entrance interacting with their mothers or outside the burrow awaiting their mother’s return. Mothers returned frequently (generally once every one to three hours) to small joeys cached in the burrows. Larger young-at-heel often accompanied their mothers as they investigated or passed by other burrows. Smaller joeys-at-heel also occasionally accompanied their mothers away from natal burrow, but always remained in close contact with their mothers, e.g., touching most of the time or under foot, unlike larger joeys-at-heel.

### Other species photographed at burrows

In addition to wombats, six reptile, 36 bird and 14 mammal species were recorded at wombat burrow entrances or entered the wombat burrow itself (Table B in S1 Appendix). Black rats (*Rattus rattus*) were the most frequently recorded species (1,839 camera events) at burrows after BNWs. Other species recorded frequently included the swamp wallaby (*Wallabia bicolor*) (607 camera events), fallow deer (*Dama dama*) (560 camera events) and wonga pigeons (*Leucosarcia melanoleuca*) (340 camera events). Eight mammal species were recorded spending more than five minutes and up to one hour inside BNW burrows including short-beaked echidna (*Tachyglossus aculeatus*), common brushtail possum (*Trichosurus vulpecula*), swamp wallaby, house mice (*Mus musculus*), black rats, feral cats (*Felis catus*) and foxes (*Vulpes vulpes*). During the pre-treatment monitoring in July 2014, a fox and two swamp wallabies were photographed that had skin lesions consistent with mange. In addition, prior to the study, a severely mange affected fox was photographed near a burrow in October 2013. At least 13 species of bird were observed foraging in the loose soil at the burrow entrance and some of these also appeared to be ingesting soil.

## Discussion

This study documents the elimination of clinical manifestations of mange from a population of BNWs, which was experiencing a mange epizootic of increasingly catastrophic proportions, using Cydectin*®* at a dosage rate of ~0.5 mg/kg administered by burrow flaps. Although apparently successful at initially eliminating mange from the Bent’s Basin wombat population, this study also showed that re-introduction of mange occurred. The use of camera traps and the marking of treated wombats, allowed us to assess the percentage of the population that was treated with each treatment period, monitor the effect of treatment on affected animals, and generate additional behavioural data on mange affected and healthy wombats that will be important to consider when designing population-wide treatments.

We believe that our successful eradication of signs of mange was largely due to attempts to place a treatment flap in front of all burrows in the treatment area and an extended treatment period (13 treatments over 24 weeks), the two most important variables identified by mathematical modelling that are critical to population-wide treatment success [18,29]. By treating all burrows, we were able to administer Cydectin*®* to the majority of the Bent’s Basin wombat population (range 74–96%) each treatment. With this degree of treatment, the number of infected wombats shedding mites and their eggs would rapidly decline and the percentage of wombats in the population susceptible to infection or re-infection (untreated wombats) would remain low.

We treated both active and inactive burrows, as our burrow monitoring showed that some wombats avoid entering burrows with treatment flaps and wombats reactivated inactive burrows. Treating all wombat burrows is challenging as some will be difficult to find and access to them may not be possible in rough terrain that is heavily vegetated. Therefore, to achieve the level of treatment application success accomplished in this study, searches for unrecognized and newly dug or re-opened burrows at each treatment are required. Lastly, the use of camera traps proved valuable in increasing the number of wombats treated, as it identified treatment flaps that required adjustments because wombats were circumventing them in their original positions.

To the authors’ knowledge, there has only been one adequately documented attempt to eradicate mange from wombat populations using burrow flaps and Cydectin*®* [29]. In that treatment attempt, all active burrows were treated, including inactive burrows that became active over time. Weekly treatment over 12 weeks, led to signs of recovery in the population and decreased prevalence over 18 weeks from commencement of treatments, but mange prevalence slowly increased in the following weeks resulting in continued decline of the population to low levels by two years post-treatment [9,16,29], persisting at low levels for years following [21,58]. The authors’ attributed this eradication failure, to a possible failure to identify all burrows, eradicate mites from all wombat burrows or all wombats, allowing disease resurgence among wombats. In subsequent analyses, the authors identified other factors that may have overcome eradication failure, such as a longer duration of treatments, a more efficacious therapeutic agent, and improved therapeutic delivery success. Based on our study, it is possible that failure to treat all burrows and those deemed to be unused, could have been the cause of continued mite presence in the environment, as we found that burrows initially deemed to be unused rapidly reverted to being used by wombats once treatment flaps were installed on active burrows. Four other treatment trials involving numbers of wombats comparable to the numbers in this current study have also been undertaken, but there is inadequate information provided on these trials to draw conclusions about their success, or on how the treatment protocols in them related to their success or failure [4,5,59,60].

In the Bent’s Basin SCA study, we monitored the resolution of clinical signs of mange during treatment and the mange status of the population after treatment using three metrics, the overall mange severity score, the individual maximum segment severity score, and the percentage of images of BNW where the animals were scratching. All three metrics showed a similar trajectory to each other and those in experimentally infected wombats [10]. Therefore, any one of these metrics could be used to monitor mange treatment success and duration. However, monitoring the percentage of images of animals scratching has an advantage, in that it is not a subjective parameter and does not require training of the observer – though caution should be used since even healthy wombats scratch.

The pattern in change in mange severity scores observed in this study and in others [10,29] suggest that an initial decrease in mange severity scores and scratching should occur by two to three weeks after the onset of treatment and the decrease in these scores will be largely reflected in a progressive detachment of crusts. Severity scores may temporarily increase again four weeks into treatment before scores decline to near zero by 12 weeks of treatment. The temporary increase in severity scores at 4 weeks after the onset of treatment reflects increasing loss of crusts and associated hair, exposing underlying healing and irritated skin. Hair regrowth will first become apparent by approximately 4 weeks after the onset of treatment; however, hair regrowth will not be extensive and easily observed in camera images until 7–8 weeks after the onset of treatment.

Other factors, in addition to the healing process occurring in the affected areas of skin, may also impact the mange severity and percentage of animals scratching in the early stages of treatment [61]. During this period, it is possible that some of the wombats with the highest severity scores die from complications of the mite infestation [4,5,8,9,16,33,61–63]. Also, by the 12th week into treatment and thereafter, mange severity scores will approach zero. However, they will never reach zero as skin injuries, such as fight wounds or hair loss from rubbing on trees or other objects, that might have a similar appearance to mange lesions will always be present in some wombats even once mange has been eliminated. Similarly, even mange-free wombats scratch, and so up to 20–25% of images of wombats in a mange-free population might be expected to show a wombat scratching.

Mange impacts wombat populations, not only by killing them, but also by decreasing reproduction rates. For example, one study [6] found that 80% of healthy female BNW had a joey-at-foot or were lactating, in contrast to 82% of mange affected BNW females showing no signs of lactation or not having joeys-at-foot. Similarly, southern hairy-nosed wombats with severe mange were found to be in a non-reproductive state [64]. Therefore, a rapid improvement in reproductive rates to normal levels [13], as observed in our study, should be expected, and can be used as another measure of successful population-wide treatment for mange.

Placing, refilling, and maintaining burrow flaps at all wombat burrow entrances came with a considerable cost in human resources. In this case, even for a relatively small population (50–60 wombats) confined to a small geographic area (50 hectares), nearly 6,000 hours of effort was required to undertake the treatment. We were able to meet our human resource needs by recruiting highly motivated volunteers from the community and partnering with a university. Treating the Bent’s Basin BNWs, however, could not be done by volunteers alone and paid staff were essential to design the treatment plan, apply for the necessary permits, analyse the data, and organise, train, and oversee the volunteers. It is likely that the number of hours required to treat a wombat population for mange could be reduced if trained consultants or staff that were familiar with the site were substituted for volunteers. While more costly, this would require less training time and smaller treatment teams. Also, although using camera traps was essential in this study for monitoring the numbers of wombats treated with each application and the success of treatment, their use may not be required in all future population treatments to the same degree or could be substituted with less labour-intensive spotlight surveys in some habitats, and this would also greatly reduce the number of person hours required. Nonetheless, we found camera surveillance enabled fine adjustment of burrow flaps to improve treatment delivery and would recommend its use where feasible.

The amount of effort required to treat a wombat population for mange could also be reduced if a medication with a longer duration of action were used and if the medication could be used with an application device that could be programmed to deliver multiple dosages over a treatment period. A recent study has shown that topical treatment with fluralaner (‘Bravecto® spot-on solution for dogs’) is safe in wombats and can maintain drug concentrations in wombats that will kill mites for at least four and possibly up to 12 weeks depending on the dosage administered [65]. Clinical resolution of sarcoptic mange was observed in all BNWs treated with Bravecto® within 3–4 weeks [65] and the authors suggest that 1–3 doses applied at monthly intervals may be all that is required to resolve mange in an individual BNW, depending on dose and disease severity [66].

Another important advancement in the treatment of mange in BNWs is a device (Marstrack™ Marsupial tracking and dosing device) currently being trialled, that can be programmed to recognize and topically treat BNW passing underneath it. This device has the capacity to deliver multiple dosages and can be programmed to treat multiple wombats that might enter or leave a burrow, and if successful, it could also represent a significant advance that will make BNW treatment more feasible. Given the propensity of wombats to destroy or dislodge burrow-flaps found in our study and others [4], regular checking of treatment devices will still be required.

Key to successful use of a multiple application device will be programming it so that it can treat as many BNWs as possible entering, leaving, or interacting with the burrow at appropriate intervals, and reducing the chances of treating the same BNWs multiple times during each treatment period. Based on our study, the optimum time for a multidose applicator device to be active would be in the two hours before and two hours after sunset, as this was the time that the bulk of BNWs in the Bent’s Basin SGA left their burrows and investigate other burrows. The treatment period, however, will likely need to be adjusted for other populations as their behaviours will likely differ to some extent [25–28,67,68]. Based on our findings, treating during the day is not indicated as females and their joey’s lounging at the burrow entrance and mangy wombats sunning themselves could be repeatedly treated. Similarly, treatment at other times at night could result in multiple dosages being administered to the same animal as females with joeys will return to the burrow regularly at night. Even with a restricted dosing interval, it is possible that wombats could be treated more than once in a night or treatment period. However, given that Cydectin® and Bravecto® have a wide therapeutic index, repeat dosing, as long as it only occurred infrequently and were not large doses, would not result in toxic overdosages [45].

Developing a device that could determine if a wombat had been treated prior to treating it again would be ideal and could prevent multiple dosing. In this study, we found that a black paint could be used to mark treated wombats and that it would fade to an undetectable level within two weeks. Therefore, by using either this black paint or another marking product co-administered with topical medication, it could be possible to develop a detection system that recognizes the marker and only applies medication to unmarked animals.

Treating joeys may not be required, as the female is the first to enter the burrow and the first to leave, none of the joeys in this study would have been directly treated by the burrow flaps. However, as moxidectin is secreted in the milk [69], a joey would therefore be dosed, at least to some extent, this way. Joeys also spent a considerable amount of time climbing around on their mother’s back and they may come in contact with topical medications applied to their mother while doing so.

Our study suggests that a dose of ~0.5 mg/kg (4 ml per BNW) of Cydectin® is all that is required to eliminate clinical signs of mange in a wombat population that is repeatedly treated using burrow flaps positioned at all known burrow entrances. The dosage we used at the time of this study was the dosage approved by an Australian Pesticide and Veterinary Medicine Act (APVMA) minor-use permit. Since then, higher dosages have been advocated, largely driven by observations made by laypeople treating individual BNW where treatments using 4 ml of Cydectin® per BNW were judged to be unsuccessful. As a result, treatments of BNW with dosages of up to 100 ml per BNW have been approved by the APVMA [43,44] and there is evidence that members of the public are using up to 200 ml of Cydectin® per BNW treatment [33]. The dosage rate of 100 ml per wombat given once a week for three weeks to two wombats with mange [42] and a single treatment with 100 ml given to nine healthy wombats [46] did not induce toxicity whilst these wombats were under observation. However, if administered through treatment flaps where repeated treatments in one night are possible, the risk of toxicity in the treated animals would be increased. Wombats administered a single 100 ml dose released a significant amount of Cydectin® in their faeces over a prolonged period [46], and coupled with an increased amount that will run off an animal when using treatment flaps, will result in increased environmental contamination [45,46]. Given these concerns and the success of our treatment of the Bents Basin population, we conclude that dosage rates exceeding 4 ml per BNW are neither required nor appropriate given the currently available data.

### Recurrence

Despite apparent success of this treatment trial at eliminating signs of mange from the Bent’s Basin population of wombats, re-infection of the population occurred. At 20-months post-treatment a single BNW with late-stage mange was photographed. This animal appeared after a severe flooding event that inundated all riverside burrows and many burrows along creek lines and therefore may have been an immigrant from an adjacent population that had been flooded out. Alternatively, it could have been a member of the Bent’s Basin population and was infected locally by some source that was not impacted by the treatment. It appears most likely that it was an immigrant as no other wombats with signs of mange were observed at that point. Nonetheless, the Bent’s Basin wombat population is not a strictly isolated population as it is possible that animals may mix with BNWs from adjacent agricultural land since BNWs may cover distances of several kilometres in a single night [53] and home ranges of up to 82.5 ha have been reported [67].

Mange had definitively re-established itself in the Bent’s Basin population by 41 months after the last treatment although the numbers of infected animals and the severity scores were significantly less than at the beginning of the treatment period. This recurrence demonstrates one of the challenges associated with population-wide treatments for mange in wombats. Showing that all potential sources of reinfection must be considered, and that if mange cannot also be eliminated in these sources, reinfection of a successfully treated population may occur, requiring regular treatment efforts at intervals as short as every two years.

Bare-nosed wombats are ecosystem engineers providing an important soil turnover and soil aeration service as well as providing shelter and denning places [70]. They also provide foraging habitats for other species and may provide mineral source sites for geophagic species such as swamp wallabies and a range of bird species [4,70,71]. The use of wombat burrows by other species, particularly sleeping chambers and burrow entrances, may create an opportunity for mange transfer to these species that could then play a role in the epizootiology of mange in wombat populations [4,5,12,29,68]. Our study provides additional data on which species use wombat modified habitats and additional evidence that burrow entrances can provide important locations for cross species *S. scabiei* transmission [30,72]. Specifically, we observed that some wombats with advanced mange regularly spent a considerable time sun baking or resting at the entrance of their burrow and therefore are likely to shed mites at burrow entrances. Considering that a significant number of different wombat individuals investigate a burrow per night, often in close succession, this means that shed mites at burrow entrances may not need to survive for extended periods off-host which may have implications for disease spread. Further research is clearly required on where mite transmission occurs and the relative frequency of transmission events in different locations (e.g., at burrow entrances, bedding chambers, and burrow tunnels).

We documented 56 species investigating, feeding, consuming earth, or loitering around the entrances to wombat burrows - eight species of mammals spent more than five minutes inside burrows, but only three species (foxes, swamp wallabies and black rats) spent an hour or longer in burrows. In Australia, foxes have previously been suggested as a vector of sarcoptic mange mites [12,64]. Pet dogs are possible mange vectors as they are highly susceptible to *S. scabiei* infection being one of the ten most common skin diseases in North American dogs [73]. Common ringtail possums, common brushtail possums, echidnas and swamp wallabies have been documented or suspected to have been infected with *S. scabiei* [2,5,74], so are possible mange vectors, though burrow visitation rates were low for all species except swamp wallabies. Cats were seen to investigate wombat burrows infrequently in this study and they are relatively resistant to sarcoptic mange, so their role in maintaining mange in BNWs would be unlikely. Black rats and fallow deer, common visitors to wombat burrow entrances in this study, are not known to be mange vectors but could potentially become transiently infected. The possible role of fallow deer should be considered as other ungulate species have been impacted by mange [75] and at least one case in fallow deer has been documented in Spain [76]. In the end, however, we provide no new evidence that other mammal species in the BNW environment played a role in this outbreak other than opportunistic observations of swamp wallabies and a fox with mange-like lesions or in the recurrence of mange, as signs of infection in them were never observed post-treatment and signs in wombats disappeared without treatment of these other species. Future studies where deer, foxes, mice and rats are culled in areas where mange is occurring in BNW to test for *S. scabiei* infection are indicated. Brushtail possums and ringtail possums could also be trapped, tested for mite infection, and released.

### Comparison of Bents Basin healthy and mange affected wombat behaviour to previous studies

Our study provides correlative support to other studies [15,27] that mange affected BNWs are more likely to be photographed scratching than healthy animals. Increased time spent scratching has been suggested to have a high energetic cost to BNWs, leading to further thermoregulation energetic costs as a result of loss of hair and skin and associated greater heat loss to the environment [15].

Similar to other studies [15,27,77], burrow emergence times for both healthy and mange affected wombats were similar and were predominately driven by seasonal changes in the time of sunset and were independent of differences in maximum daily temperature between seasons. It has been suggested that BNWs with mange may spend more time away from their burrows during the day feeding in an apparent attempt to compensate for their increased energy demands [50]. At Bents Basin this was manifested by a later return to the burrow by wombats with late-stage mange. We also detected changes in the behaviour of BNWs with severe mange, but most appeared to maximize exposure to radiant energy from the sun and the environment or to avoid feeding during the coolest part of the night, making multiple foraging bouts per night. We recorded proportionally more diurnal activity at the burrow entrances by mange affected animals than by healthy animals. Much of this activity was sunbaking (warming behaviour), however, some of the diurnal activity in severely affected mangy wombats may have been driven by the need to spend more time attempting to feed, as feeding behaviour has been demonstrated to be less efficient with mangy wombats engaging in shorter grazing bouts and increased inactivity bouts when foraging [16] and showed slower feeding rates [15]. Feeding late in the day may also benefit wombats with advanced mange as they would be exposed to sun and be out of their cooler burrows during the hottest part of the day as alopecia-impacted mangy wombats have been demonstrated to lose 1.56 to 5.88 times more energy through heat loss than healthy wombats [16].

More severely affected BNWs also spent less time away from their burrows between midnight and sunrise avoiding the coldest part of the day in the relative warmth of their burrows. However, severely mange affected BNWs did feed late into early morning in some instances, and they were more likely to return to their burrows later than healthy wombats, when the ambient air temperature was higher and this might also represent an attempt to warm themselves before entering their burrow. We suspect, but have inadequate data to determine, that BNWs with more severe mange, undertook a greater number of foraging bouts per night as we regularly recorded mange affected BNWs returning to the burrow around midnight for several hours before exiting again.

BNWs regularly switch burrows [27], and the frequency of burrow switching and the probability that the previous occupant may have had mange may play an important role in the speed of development of a mange epizootic and should influence treatment design. During our study, healthy and mange affected BNWs occupied burrows for between 1 and 7 consecutive days before switching burrows, similar to the range reported in other studies [25,27,28]. During treatment periods T01 to T05, in contrast to a previous study [27], we found that mange affected BNWs switch burrows more frequently than healthy BNWs. We also found that the proportion of burrows occupied by a healthy BNW directly after a BNW with mange increased as the prevalence of mange increased, thereby increasing the risk of *S. scabiei* transmission as the prevalence of mange in a population increases.

BNWs are known to simultaneously share a burrow with one or more BNWs, although this may reflect use of different tunnels and bedding chambers by each individual [30]. How often this occurs and how many wombats might share a burrow at the same time, appears to vary between populations and may be related to population density. The number of BNW synchronously sharing a burrow can range from 2 to 5 [26,28] with up to 71% of burrows containing multiple wombats [26]. Knowing the frequency of burrow sharing and numbers of BNW’s per shared burrow are important variables to consider when designing a mange treatment regimen. In our study, we also observed burrow sharing on at least ten occasions where two to three adult BNWs synchronously occupied the same burrow.

## Conclusion

Our study shows that treatment of an isolated population of BNWs for mange can successfully be done using a Cydectin® dose of ~0.5 mg/kg applied by burrow flaps, providing that all active, inactive and newly dug burrows are treated using recommended treatment frequencies and durations. Our study provides evidence that much higher dosages advocated by some are not required.

Based on our study, successful treatment of a bare-nosed wombat population, however, may require a large effort, possibly up to 100 person hours per wombat treated. In the future, the use of devices that can be programmed to administer multiple dosages of a long-acting mange treatment, may be critical to making population scale treatment of BNWs more feasible. Our study adds to the weight of evidence that healthy and mange affected BNW behaviours are similar across much of their range. However, there are significant differences as well, and adjusting treatment approaches to these differences could potentially impact their success. Our study also suggests, that even under ideal circumstances, reappearance of mange in a treated population is likely, requiring periodic re-treatment. Our observed use of wombat burrows and their entrances by wombats with mange and other species of mammals suggest that additional study is needed into the role of these other species in the epizootiology of mange in Australian wildlife.

## Supporting information

S1 Appendix Supporting tables and figures. (DOCX)

S2 Tables Supporting data. (XLSX)
