# Supplementary material for: Treatment and monitoring of a high-density population of bare-nosed wombats for sarcoptic mange
Source: PLoS One. 2025 Oct 1;20(10):e0332138. doi: 10.1371/journal.pone.0332138 (PMC12488012; doi:10.1371/journal.pone.0332138)
Supplement: S1 Appendix — (DOCX) [file pone.0332138.s001.docx]

**Supporting Tables:**

**S1 Table A.** **Additional variables derived from each camera event.**

| **Variable** | **Description of variable** |
| --- | --- |
| **Number of animals** | Number of individual BNWs in the camera event. |
| **Presence of young** | Presence of large pouch young or joey-at-heel – Yes / No. |
| **Number of burrow flap interactions** | Number of times the treatment flap was lifted such that a treatment would have been administered (if still present in the reservoir). Interactions included animals that entered or exited the burrow as well as animals that stood just inside the burrow entrance. When no treatment flap was present (during post-treatment monitoring), this was calculated as the number of times the head / shoulders of a BNW entered / exited the burrow entrance. |
| **First animal through the flap** | Yes / No. Yes - indicates the BNW would have been treated, i.e., the first animal through the flap after the treatment reservoir was filled / refilled. |
| **Marked with black paint** | Yes (darkly marked); Yes (faintly marked); No; and Unknown - not enough of the BNW visible to determine. |
| **Animal scratching** | Yes / No |

**S1 Table B. Number of camera events of species other than BNWs at burrows.**

^*^ = species observed remaining in burrow for > 5 minutes; ^#^ = species observed foraging in burrow entry or consuming soil/grit/minerals from burrow entrance.

| **Common name** | **Scientific name** | **Number of camera events** |
| --- | --- | --- |
| **Reptiles** |  |  |
| Bar-sided skink | *Cocinnia tenuis* | 3 |
| Eastern water dragon* | *Intellagama lesueurii lesueurii* | 229 |
| Bearded dragon | *Pogona barbata* | 4 |
| Lace monitor | *Varanus varius* | 22 |
| Red-bellied black snake | *Pseudechis porphyriacus* | 7 |
| Eastern brown snake | *Pseudonaja textilis* | 2 |
| **Birds** |  |  |
| Bar-shouldered dove^#^ | *Geopelia humeralis* | 40 |
| Wonga pigeon^#^ | *Leucosarcia melanoleuca* | 340 |
| Common bronzewing^#^ | *Phaps chalcoptera* | 88 |
| White-faced heron | *Egretta novaehollandiae* | 4 |
| Eurasian coot | *Fulica atra* | 5 |
| Dusky moorhen | *Gallinula tenebrosa* | 5 |
| Purple swamphen | *Porphyrio porphyrio* | 3 |
| Azure kingfisher | *Ceyx azureus* | 4 |
| Laughing kookaburra | *Dacelo novaeguineae* | 1 |
| Satin bowerbird | *Ptilonorhynchus violaceus* | 43 |
| Superb fairy-wren^#^ | *Malurus cyaneus* | 129 |
| Variegated fairy-wren^#^ | *Malurus lamberti* | 5 |
| Unidentified fairy-wren^#^ | *Malurus* sp. | 109 |
| Brown thornbill | *Acanthiza pusilla* | 4 |
| White-throated gerygone^#^ | *Gerygone olivacea* | 26 |
| White-browed scrubwren^#^ | *Sericornis frontalis* | 96 |
| Weebill | *Smicrornis brevirostris* | 2 |
| Spotted pardalote^#^ | *Pardalotus punctatus* | 3 |
| Eastern spinebill | *Acanthorhynchus tenuirostris* | 6 |
| Noisy miner | *Manorina melanocephala* | 2 |
| Bell miner | *Manorina melanophrys* | 1 |
| Lewin's honeyeater | *Meliphaga lewinii* | 27 |
| Eastern whipbird^#^ | *Psophodes olivaceus* | 56 |
| Grey shrike-thrush^#^ | *Colluricincla harmonica* | 44 |
| Olive-backed oriole | *Oriolus sagittatus* | 6 |
| Grey butcherbird | *Cracticus torquatus* | 1 |
| Australian magpie | *Gymnorhina tibicen* | 4 |
| Pied currawong | *Strepera graculina* | 9 |
| Grey fantail | *Rhipidura albiscapa* | 4 |
| Willie wagtail | *Rhipidura leucophrys* | 1 |
| Rufous fantail | *Rhipidura rufifrons* | 1 |
| Australian raven | *Corvus coronoides* | 3 |
| Eastern yellow robin^#^ | *Eopsaltria australis* | 103 |
| Eurasian blackbird^#^ | *Turdus merula* | 256 |
| Bassian thrush^#^ | *Zoothera lunulata* | 2 |
| Silvereye | *Zosterops lateralis* | 4 |
| Red-browed finch | *Neochmia temporalis* | 6 |
| Unidentified birds |  | 15 |
| **Mammals** |  |  |
| Short-beaked echidna* | *Tachyglossus aculeatus* | 60 |
| Common ringtail possum | *Pseudocheirus peregrinus* | 50 |
| Common brushtail possum* | *Trichosurus vulpecula* | 161 |
| Eastern grey kangaroo | *Macropus giganteus* | 7 |
| Swamp wallaby*# | *Wallabia bicolor* | 607 |
| Common wallaroo | *Osphranter robustus* | 3 |
| House mouse* | *Mus musculus* | 4 |
| Black rat^*^ | *Rattus rattus* | 1,839 |
| Unidentified Rattus species^*^ | *Rattus* sp. | 231 |
| Dog | *Canis familiaris* | 7 |
| Fox^*^ | *Vulpes vulpes* | 145 |
| Cat^*^ | *Felis catus* | 45 |
| Rabbit^*^ | *Oryctolagus cuniculus* | 1 |
| Brown hare | *Lepus capensis* | 3 |
| Fallow deer | *Dama dama* | 560 |
| Unidentified mammals |  | 51 |

**Supporting figures**:


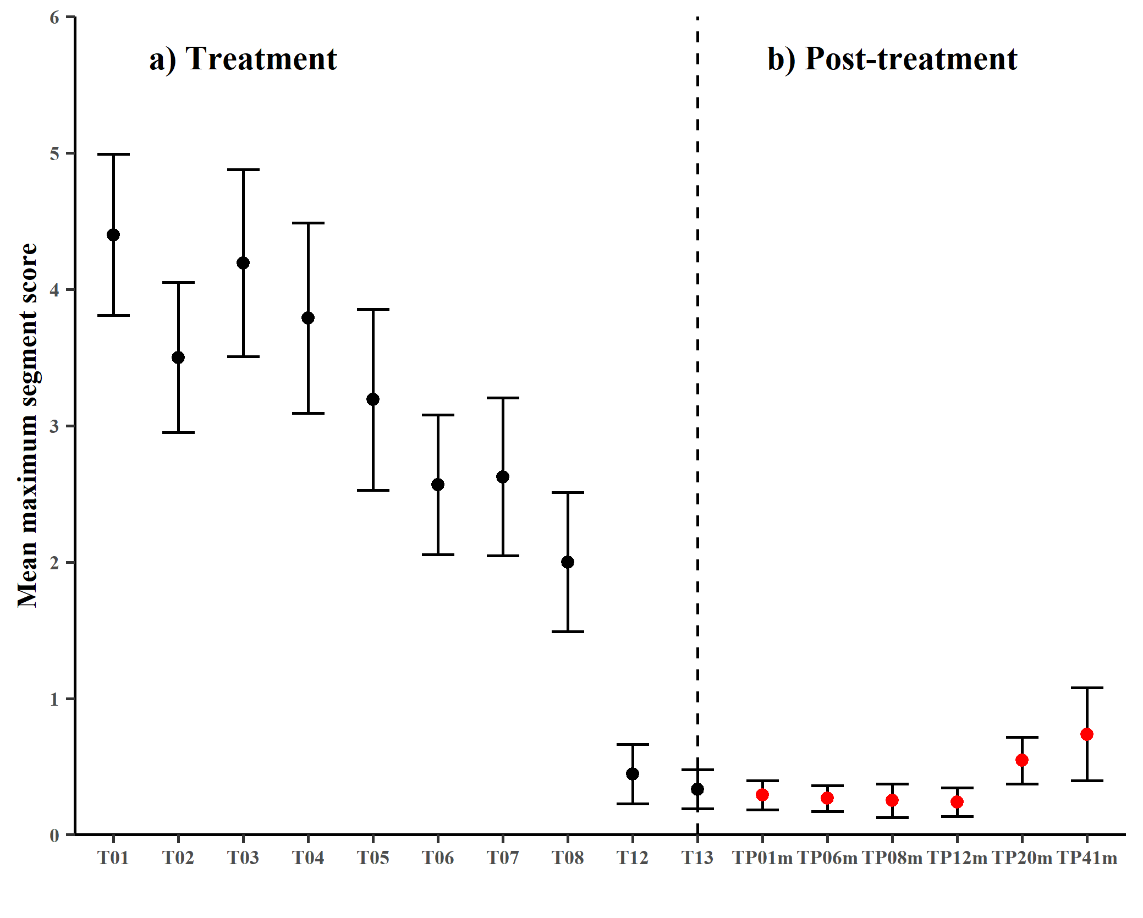


**S1 Fig A.** **Mean maximum segment score of first animal through the treatment flap -** a) Treatment (closed black circles) and b) post-treatment (closed red circles). Error bars are ± 1 standard error.


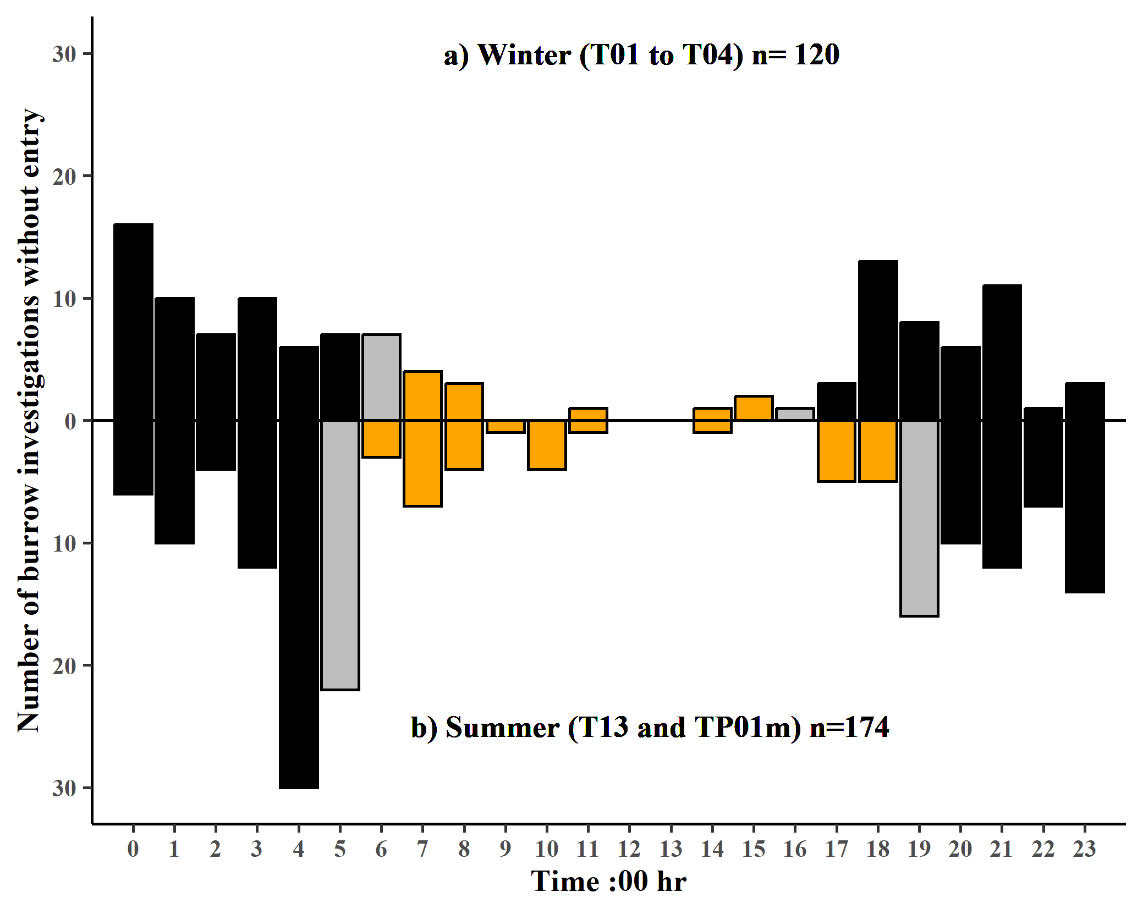


**S1 Fig B.** **Frequency distribution of camera events showing burrow investigation without entry with time of day in winter and in summer.** Data is pooled for 4 weeks of monitoring. a) Upper panel - winter (T01 to T04 pooled) and b) Lower panel - summer (T13 and TP01m pooled). Bars represent one-hour increments and n = the number of camera events of burrow investigation without entry. Orange bars represent burrow investigations without entry during daylight hours, grey bars are the hour increment in which sunrise or sunset occurred and black bars are night-time investigations without entry.
